# Human Platelet Lysate Maintains Stemness of Umbilical Cord-Derived Mesenchymal Stromal Cells and Promote Lung Repair in Rat Bronchopulmonary Dysplasia

**DOI:** 10.3389/fcell.2021.722953

**Published:** 2021-11-10

**Authors:** Guilian Liao, Yan Liao, Duanduan Li, Zeqin Fu, Shiduo Wu, Danling Cheng, Qiuxing Ouyang, Zan Tang, Guifang Zeng, Xiao Liang, Shaokun Xu, Junyuan Hu, Muyun Liu

**Affiliations:** ^1^Obstetrics and Gynecology, Maternal and Child Health Hospital of Longgang District, Shenzhen, China; ^2^Shenzhen Beike Biotechnology Co., Ltd., Shenzhen, China; ^3^Neurological Rehabilitation for Children, Maternal and Child Health Hospital of Luohu District, Shenzhen, China; ^4^National-Local Associated Engineering Laboratory for Personalized Cell Therapy, Shenzhen, China

**Keywords:** mesenchymal stromal cells, human platelet lysate, bronchopulmonary dysplasia, senescence, lung repair

## Abstract

Mesenchymal stromal cells (MSCs) show potential for treating preclinical models of newborn bronchopulmonary dysplasia (BPD), but studies of their therapeutic effectiveness have had mixed results, in part due to the use of different media supplements for MSCs expansion *in vitro*. The current study sought to identify an optimal culture supplement of umbilical cord-derived MSCs (UC-MSCs) for BPD therapy. In this study, we found that UC-MSCs cultured with human platelet lysate (hPL-UCMSCs) were maintained a small size from Passage 1 (P1) to P10, while UC-MSCs cultured with fetal bovine serum (FBS-UCMSCs) became wide and flat. Furthermore, hPL was associated with lower levels of senescence in UC-MSCs during *in vitro* expansion compared with FBS, as indicated by the results of β-galactosidase staining and measures of senescence-related genes (CDKN2A, CDKN1A, and mTOR). In addition, hPL enhanced the proliferation and cell viability of the UC-MSCs and reduced their doubling time *in vitro*. Compared with FBS-UCMSCs, hPL-UCMSCs have a greater potential to differentiate into osteocytes and chondrocytes. Moreover, using hPL resulted in greater expression of Nestin and specific paracrine factors (VEGF, TGF-β1, FGF2, IL-8, and IL-6) in UC-MSCs compared to using FBS. Critically, we also found that hPL-UCMSCs are more effective than FBS-UCMSCs for the treatment of BPD in a rat model, with hPL leading to improvements in survival rate, lung architecture and fibrosis, and lung capillary density. Finally, qPCR of rat lung mRNA demonstrated that hPL-UCMSCs had lower expression levels of inflammatory factors (TNF-α and IL-1β) and a key chemokine (MCP-1) at postnatal day 10, and there was significant reduction of CD68^+^ macrophages in lung tissue after hPL-UCMSCs transplantation. Altogether, our findings suggest that hPL is an optimal culture supplement for UC-MSCs expansion *in vitro*, and that hPL-UCMSCs promote lung repair in rat BPD disease.

## Introduction

Bronchopulmonary dysplasia (BPD) is a serious and common complication of prematurity (Jobe and Bancalari, 2001; Kinsella et al., 2006), which requires mechanical ventilation and oxygen therapy (Baraldi and Filippone, 2007). It is characterized by a prominent inflammatory response in the lungs that causes restricted lung growth (tissue simplification), subdued alveolar and blood vessel development, and dramatically impaired pulmonary function (Khemani et al., 2007; del Cerro et al., 2014). Preventive approaches, including alternative ventilator strategies and use of corticosteroids (an anti-inflammatory medication), have limited success and unacceptable side effects (Baveja and Christou, 2006). Therefore, the need for new therapies is urgent.

Recent advances in stem cell research hold promise for the prevention and treatment of a range of chronic debilitating diseases. Previous research has investigated the effectiveness of mesenchymal stem/stromal cells (MSCs)-based therapies for ameliorating lung injury associated with preterm birth (Aslam et al., 2009; van Haaften et al., 2009; Willis et al., 2018). MSCs have been shown to differentiate into a variety of tissue cell types, including endothelial cells and specific lung cells (Rojas et al., 2005; Phinney and Prockop, 2007). A growing number of studies in pulmonary medicine have demonstrated that MSC therapy can ameliorate bleomycin, endotoxin, lipopolysaccharide (LPS), or hyperoxia-induced lung injury through the paracrine pathway. These studies demonstrate that MSCs can be used to repair damaged tissue and deliver protection *via* the secretion of specific growth and immunoprotective factors (Ortiz et al., 2003; Gupta et al., 2007; Mei et al., 2007; Xu et al., 2007; Willis et al., 2018).

Various sources of MSCs have been extensively explored in the context of BPD therapy development, including human umbilical cord (hUC) (Chaubey et al., 2018; Willis et al., 2018), human umbilical cord blood (hUCB) (Chang et al., 2014; Kwon et al., 2019), and bone marrow (BM) (Aslam et al., 2009; van Haaften et al., 2009). hUC-derived MSCs (hUC-MSCs) are particularly useful in clinical application because they are easy to obtain, more proliferative, and have more powerful paracrine function than other sources (Wu et al., 2020; ClinicalTrials.gov: NCT01207869 and NCT02443961). Specially, secretome studies of MSCs isolated from different tissue sources were reported by Konala et al. (2020), and gene expression analysis showed the increased expression of FGF2, PDGF-1, VEGF, and IL-6 in Wharton’s jelly MSCs (WJ-MSCs) over BM-MSCs. Arrigoni et al. (2020) demonstrated that the higher production of TGF-β, chemokines and anti-inflammatory cytokines could make the WJ-MSCs’ secretome a perfect candidate to control the inflammatory process, rather than hUCB-MSCs or BM-MSCs. However, even when using the same source and complying with good manufacturing practice (GMP) requirements, the protocols currently in use for MSC production are variable (Ikebe and Suzuki, 2014). Small variation in the processing methods, such as the type of culture media used, may have significant effects on the final characteristics and functionality of the MSC-based product (Panchalingam et al., 2015). The majority of human MSCs that have been used in preclinical and clinical studies of BPD were expanded *in vitro* in Dulbecco’s Modified Eagle’s Medium (DMEM), supplemented with 10 or 20% fetal bovine serum (FBS) (Chang et al., 2014; Liu et al., 2014; Reiter et al., 2017). Although FBS has long been employed as a widely accepted standard cell culture supplement for both research and clinical use (Wu et al., 2017), FBS may not be an effective enough culture for clinical applications of human MSCs, especially because FBS culture use is characterized by the addition of xenogeneic proteins, increased immunogenicity and the promotion of cell aging (Trubiani et al., 2015). Søndergaard et al. (2017) suggested that the low proliferation rates of FBS-expanded adipose-derived stromal cells (ASCs) could be signs of senescence or quiescence. As an alternative to FBS, a commercially available human platelet lysate (hPL) has been used recently as a culture supplement to promote MSC growth (Griffiths et al., 2013). hPL use enhanced the proliferation rate of ASCs compared with FBS at normoxia (Søndergaard et al., 2017). The umbilical cord matrix (UCM)-derived MSCs (UCM-MSCs) cultured under static conditions using hPL-supplemented medium expanded more rapidly compared with UCM-MSCs that were expanded using a previously established protocol (de Soure et al., 2017). Of course, many other factors also affect the characteristics and function of MSCs, such as the biomaterials, that were used to support the growth of stem cells, or the role of environment in MSCs’ behavior. Tatullo et al. (2019) demonstrated that the innovative combination of PLA-Based Mineral-Doped Scaffolds colonized with MSCs from periapical cyst represent a promising strategy in tissue engineering and organs repair, it promotes cell proliferation, cell viability, and gene expression for osteogenic and odontogenic differentiation (Tatullo et al., 2019). More interestingly, the multipotency of scaffolds is a new concept. It is highlighting the importance of the environment in directing MSCs homing and differentiation (Aulino et al., 2015).

In the current study, we will use a neonatal rat BPD model to evaluate the therapeutic effects of UC-MSCs that were cultured with FBS versus hPL as the growth supplement. The saccular and alveolar stages of lung development in the rat occur around the time of birth with alveolarization starting on postnatal day 5 and continuing up to 2 weeks of age. Hence, the developmental stage of the rat lung at birth resembles that of the human preterm neonate at between 24- and 28-weeks’ gestation, making the newborn rat an excellent model to study human developmental lung injury. Indeed, hyperoxia-induced lung injury in neonatal rat is similar to BPD, with rarification and simplification of alveoli, thickened alveolar septa, and right ventricular hypertrophy (Hansmann et al., 2012). In the current study, we provide evidence that hPL is useful for UC-MSCs expansion and rejuvenation *in vitro*, and has superior therapeutic efficacy compared to FBS in an *in vivo* rat BPD model. Our results will help support future successful application of UC-MSCs for the treatment of BPD infant patients.

## Materials and Methods

### Animals

Timed pregnant Wistar rats were purchased from the Animal Center at the Nanfang Medical School, Guangdong Province, China. All rats were maintained in a specific pathogen-free facility, and all animal procedures and protocols were reviewed and approved by the Animal Care and Use Committee of Shenzhen Beike Biotechnology Co., Ltd.

### Cell Isolation and Culture

Human umbilical cord-derived mesenchymal stromal cells were isolated following a previously reported process (Willis et al., 2018; Liu et al., 2020). Briefly, the umbilical cord was obtained from a healthy pregnant woman after she provided informed consent. The umbilical cord was rinsed twice with Dulbecco’s Phosphate-Buffered Saline (D-PBS, Invitrogen), cut longitudinally, and the arteries and veins were removed. The soft gel tissues were dissected into small pieces and individually placed on 100-mm tissue culture dishes with DMEM (Gibco) supplemented with 10% (v/v) FBS (Hyclone). After 12 days of culture, umbilical cord tissue was carefully removed. Plates were washed three times with D-PBS; the plastic adherent cell colonies were trypsinized and maintained in culture in DMEM complete medium with 10% FBS (Passage 0, P0). From P1 to P10, UC-MSCs were divided into two groups that were cultured with 10% (v/v) FBS or 5% (v/v) hPL (UltraGRO^TM^-Advanced, GMP Grade, AventaCell BioMedical), as well as 2 mm L-glutamine, and 1% penicillin/streptomycin. UltraGRO^TM^-Advanced is a cell culture supplement derived from human single donor platelets collected from healthy donors at FDA-licensed centers, and each donor has been tested using FDA-licensed tests. In our experiments, The Lot No. of UltraGRO^TM^-Advanced (GMP Grade) we used is 7AHF18G. Both groups were incubated at 37°C in a humidified atmosphere of 5% CO_2_.

### Cell Characterization and Differentiation

Cytometric evaluation of UC-MSCs’ surface and intracellular profiles was carried out at P4. Antibodies used for cytometric analysis were CD90-FITC (5E10), CD105-APC (266), CD73-PE (TY/23), CD34-PE (563), CD45-FITC (HI30), HLA-DR-PerCP (G46-6), and Nestin-APC (196908) along with the corresponding isotype control antibodies. All the antibodies were purchased from BD Pharmingen (San Diego, CA, United States) or R&D Systems (Abingdon, United Kingdom). Flow cytometry was performed using a BD^TM^ Aria IIu flow cytometer, and the data were analyzed with the FlowJo 7.5 software (Treestar, Ashland, OR, United States).

For osteogenic, adipogenic, and chondrogenic differentiations of MSCs *in vitro*, FBS-UCMSCs and hPL-UCMSCs were cultured in the relevant differentiation media for 2–3 weeks and analyzed by staining with Alizarin Red, Oil Red O, and toluidine blue staining, as previously described (Liao et al., 2020).

### Umbilical Cord-Derived Mesenchymal Stromal Cells Proliferation Assay

Fetal bovine serum umbilical cord-derived mesenchymal stromal cells and hPL-UCMSCs were resuspended in DMEM complete medium (supplemented with 10% FBS) and seeded to a 12-well plate at 10^4^ cells per well. The cells were trypsinised at each indicated time point over 5 days, and cell numbers were counted directly. Population doubling times (DTs) of UC-MSCs were calculated at P4 using the following formula: DT = *t* × [log 2/(log *N*_*t*_−log*N*_0_)], where *N*_*t*_ is the number of harvested cells, *N*_0_ is the number of seeded cells and t is the culture time.

### Experimental Design of Rat Bronchopulmonary Dysplasia Model

Newborn rat pups (∼50 L) were randomly allocated to five experimental groups: normoxia control group (NRMX), hyperoxia BPD group (HYRX), hyperoxia with saline treatment (HYRX + Saline group), hyperoxia with hPL-UCMSCs treatment (HYRX + hPL-UCMSCs group), and hyperoxia with FBS-UCMSCs treatment (HYRX + FBS-UCMSCs group). Litters for each experimental group were limited to 10 pups to control for the effect of litter size on nutrition and growth.

In detail, newborn rats, along with their mother, were exposed to either hyperoxia (95% O_2_) or normoxia (room air, 21% O_2_) from birth to postnatal day 10 (PN10) in cages in an airtight Plexiglas chamber. Mothers were rotated from hyperoxia to normoxia every 24 h to minimize excessive oxygen toxicity to the adult animals. Standard rat pellet diet and water were provided *ad libitum*. In treatment groups, newborn rats were injected intratracheally with 50 μl saline with or without UC-MSCs (FBS-UCMSCs or hPL-UCMSCs, 5.0 × 10^5^ cells/per animal) at PN4. At PN10, newborn rats were sacrificed and the whole lung tissue was harvested for histology (hematoxylin–eosin, H&E, and Masson’s trichrome staining), total lung mRNA analysis, and immunohistochemistry.

### Assessment of Umbilical Cord-Derived Mesenchymal Stromal Cells Engraftment *in vivo*

To analyze the biodistribution of UC-MSCs in lung, 5.0 × 10^5^ fluorescent dye-labeled FBS-UCMSCs or hPL-UCMSCs were administrated through intratracheal injection at PN4. The *in vivo* tracing of cells at different time points (PN4, PN6, PN8, and PN10) was detected using an *in vivo* imaging system (Berthold, LB983NC100).

### Lung Tissue Collection

Following anesthesia with 60 mg/kg pentobarbital (injected intraperitoneally, i.p.), lungs were perfused with PBS through the right ventricle (RV). The right lung was removed and lysed in TRIzol reagent. The left lung was inflated to a fixed pressure of 20 cm H_2_O with 4% paraformaldehyde (PFA) *in situ* and stored in 4% PFA overnight. One part of each fixed left lung tissue was transferred to 75% ethanol before subsequent processing, and was then paraffin embedded for sectioning, sectioned at 5-μm, and stained with H&E. The other part of each fixed left lung tissue was dehydrated with 30% sucrose for 24 h, then cut into 5-μm frozen sections and stored at −80°C.

### Lung Parenchymal, Pulmonary Vascular Morphometry, Immunofluorescence, and Immunohistochemistry

Following lung fixation, lung sections were analyzed for histology. Lung sections were stained with H&E and Masson’s Trichrome (collagen deposition). Randomly selected areas (five fields) from 5-μm thick lung sections were captured at 100× (H&E) and 200× (Masson’s Trichrome) magnification using a Leica DM500B microscope (Leica, Germany). Large airways and vessels were avoided for the lung morphometry. To measure mean linear intercept (MLI), a grid with parallel lines spaced at 60 μm was overlaid onto the image, and the length of each chord, defined by the intercept with alveolar walls, was recorded. The degree of collagen deposition was measured using ImageJ software and expressed as percentage of collagen deposition per total septal area. The lung microvasculature was determined by counting the number of von Willebrand factor (vWF)-positive vessels (diameter <50 μm) in five random images at 100× magnification, and the number of macrophages in lung tissue was counted by measuring CD68-positive cells in five random high fields. Tissue sections were incubated with primary antibody (vWF, 1:400; CD68, 1:200, Abcam). Afterward, slides were washed and incubated with Alexa Fluor 488-congugated donkey anti-rabbit (1:500) antibody.

### Reverse Transcription and Real-Time qPCR

Total RNA was extracted from lung tissues using the TRIzol reagent (Invitrogen), and 1 μg of RNA was reverse transcribed using a RevertAid First Strand cDNA Synthesis Kit (Thermo Scientific). The generated cDNA was subjected to real-time PCR with the SYBR Green reagent (Roche) using the following human and rat primers listed in Tables 1, 2. The relative mRNA abundances were calculated using the ΔCt methods, and the gene expression levels were normalized with respect to those of GAPDH.

**TABLE 1:** Primers used for the amplification of human transcripts by real-time quantitative PCR.

Genes	Forward sequence (5′–3′)	Reverse sequence (5′–3′)
GAPDH	GTCTCCTCTGACTTCAACA GCG	ACCACCCTGTTGCTGTAGC CAA
CDKN1A (p21)	AGGTGGACCTGGAGACTCT CAG	TCCTCTTGGAGAAGATCAG CCG
CDKN2A (p16^INK4A^)	CTCGTGCTGATGCTACTGA GGA	GGTCGGCGCAGTTGGGC TCC
P53	CCTCAGCATCTTATCCGAG TGG	TGGATGGTGGTACAGTCA GAGC
mTOR	AGCATCGGATGCTTAGGAG TGG	CAGCCAGTCATCTTTGGAG ACC
HGF	GAGAGTTGGGTTCTTACTGC ACG	CTCATCTCCTCTTCCGTGG ACA
EGF	TGCGATGCCAAGCAGTCTG TGA	GCATAGCCCAATCTGAGAAC CAC
IGF1	CTCTTCAGTTCGTGTGTGGA GAC	CAGCCTCCTTAGATCACAG CTC
VEGF	TTGCCTTGCTGCTCTACCT CCA	GATGGCAGTAGCTGCGCTG ATA
TGF-β1	TACCTGAACCCGTGTTGCT CTC	GTTGCTGAGGTATCGCCAG GAA
FGF2	AGCGGCTGTACTGCAAAAA CGG	CCTTTGATAGACACAACTCCT CTC
IL-6	AGACAGCCACTCACCTCTT CAG	TTCTGCCAGTGCCTCTTTG CTG
IL-8	GAGAGTGATTGAGAGTGGAC CAC	CACAACCCTCTGCACCCAG TTT

**TABLE 2:** Primers used for the amplification of rat transcripts by real-time quantitative PCR.

Genes	Forward sequence (5′–3′)	Reverse sequence (5′–3′)
GAPDH	CTCTACCCACGGCAAGTTCAA	GGGATGACCTTGCCCACAGC
TNF-α	TACTGAACTTCGGGGTGATCG GTCC	CAGCCTTGTCCCTTGAAGAG AACC
IL-1β	AAATGCCTCGTGCTGTCTGA	TGGAGAATACCACTTGTTGGCT
TGF-β	CAACTGTGGAGCAACACGTAGA	CAACCCAGGTCCTTCCTAAAGT
VEGF	CTGCTCTCCTGGGTGCATTG	ACTCCTGGAAGATGTCCACCA
MCP-1	ATGCAGTTAATGCCCCAGTCA	TTCTCCAGCCGACTCATTGG

### Statistical Analysis

All results represent at least three independent experiments and are expressed as mean ± SEM. All statistical comparisons were made using a two-tailed Student’s *t*-test (between two groups) or one-way ANOVA (for multi-group comparisons). *p* < 0.05 was considered significant. Analysis and graphing were performed using the Prism software (v 5.01, GraphPad).

## Results

### Rejuvenation of Human Umbilical Cord-Derived Mesenchymal Stromal Cells Cultured With Growth Supplement Human Platelet Lysate

**FIGURE 1:**
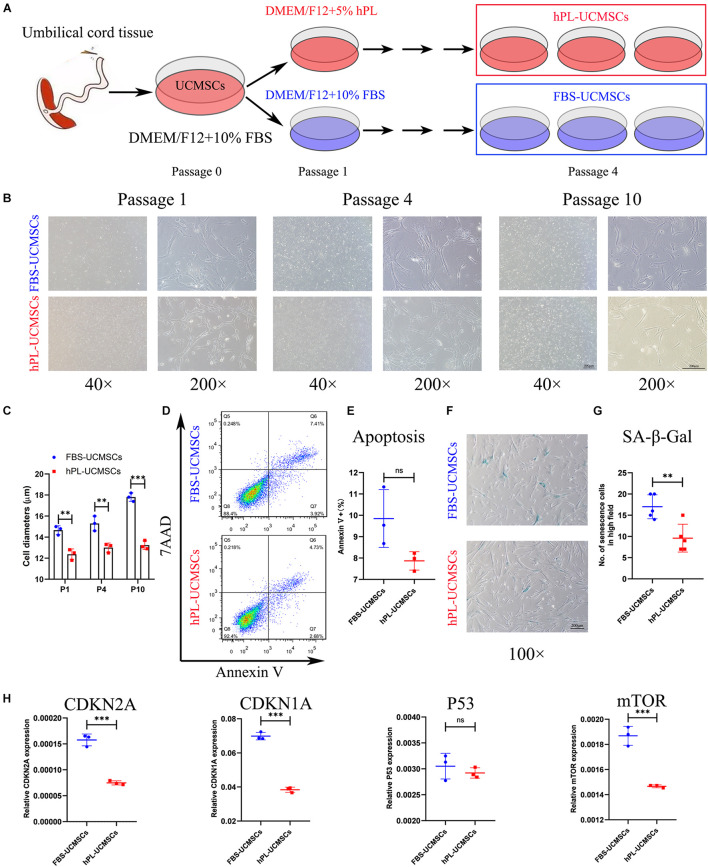
Growth supplement hPL could largely reduce the senescence of UC-MSCs *in vitro* expansion compared with FBS. **(A)** Schematic of protocols used for UC-MSCs *in vitro* expansion with either FBS-supplemented (10% FBS) or hPL-supplemented (5% hPL) media for up to 10 passages. Representative images showing the morphology of UC-MSCs changes notably as a result of serial passaging (from P1 to P10) between the FBS- and hPL-supplemented groups **(B)**; and the cell diameters in both groups were measured **(C)**. Scale bars, 100 μm (40×) or 200 μm (200×). At Passage 10, the cell apoptosis of FBS-UCMSCs and hPL-UCMSCs were analyzed using flow cytometry by staining with Annexin V and 7AAD reagent **(D)**; and the percentage of Annexin V^+^ cells was compared between these two groups **(E)**. At Passage 10, the cell senescence of FBS-UCMSCs and hPL-UCMSCs were analyzed by staining with β-galactosidase reagent **(F)**. Scale bars, 200 μm; and the count of SA-β-Gal positive cells in high field (100×) was compared **(G)**. **(H)** The mRNA expressions of senescence-related gene (CDKN2A, CDKN1A, P53, and mTOR) in P10 FBS-UCMSCs and hPL-UCMSCs were analyzed by qPCR. Data are shown as mean ± SEM. *n* = 3–5. ^∗∗^*p* < 0.01, ^∗∗∗^*p* < 0.001. ns, not significant.

Mesenchymal stromal cells are prone to senescence *in vitro* after serial passage. The adjustment of growth supplement type is an important issue in the optimization of the stem cell manufacturing protocol. In our study, all primary UC-MSCs (P0) were first cultured in DMEM/F12 with 10% FBS, and then divided into two groups with different medium supplement for culturing from P1 to P10, either 10% FBS (FBS-UCMSCs group) or 5% hPL (hPL-UCMSCs group) (Figure 1A). For both groups, P1, P4, and P10 MSCs were used for showing their morphology, P10 cells were used for apoptosis and senescence analyses, and P4 cell populations were mainly used for the following *in vitro* and *in vivo* experiments.

The characteristics of cell morphology, cell apoptosis, cell senescence, and cell growth were examined for hPL-UCMSCs and FBS-UCMSCs. First, P1 UC-MSCs from both culture conditions exhibited a similar fibroblast-like morphology, but hPL-UCMSCs appeared slightly smaller than FBS-UCMSCs (Figure 1B); we also found that from P4 to P10 the majority of FBS-UCMSCs gradually became enlarged and flat, while hPL-UCMSCs maintained their small size and well-formed structure across the culture period, and their cell diameters from P1 to P10 were measured (Figures 1B,C). Next, cell apoptosis and senescence of P10 UC-MSCs were assessed using flow cytometry and SA-β-Gal staining. The results of flow cytometry showed that at P10, the proportion of Annexin V^+^ cells was numerically lower for hPL-UCMSCs than FBS-UCMSCs (7.87 ± 0.35 versus 9.85 ± 1.10%), but this difference was not significant (*p* = 0.073, Figures 1D,E). Additionally, FBS-UCMSCs exhibited more SA-β-Gal positive cells (shown as blue cells) than hPL-UCMSCs, indicating higher levels of cell senescence (Figure 1F); this result was confirmed by a group comparison of the number of senescent cells, which was lower for the hPL-UCMSCs group compared with the FBS-UCMSCs group (9.60 ± 2.94 versus 17.00 ± 2.53, Figure 1G). Next, we employed qPCR to assess the expression of senescence-related genes (CDKN2A, CDKN1A, P53, and mTOR) by the hPL-UCMSCs and FBS-UCMSCs at P10. The results showed that hPL-UCMSCs expressed lower levels of CDKN2A, CDKN1A, and mTOR than FBS-UCMSCs, but P53 expression was similar (Figure 1H).

### Maintenance of Stem Cell Characteristics for Human Umbilical Cord-Derived Mesenchymal Stromal Cells Cultured With Human Platelet Lysate

**FIGURE 2:**
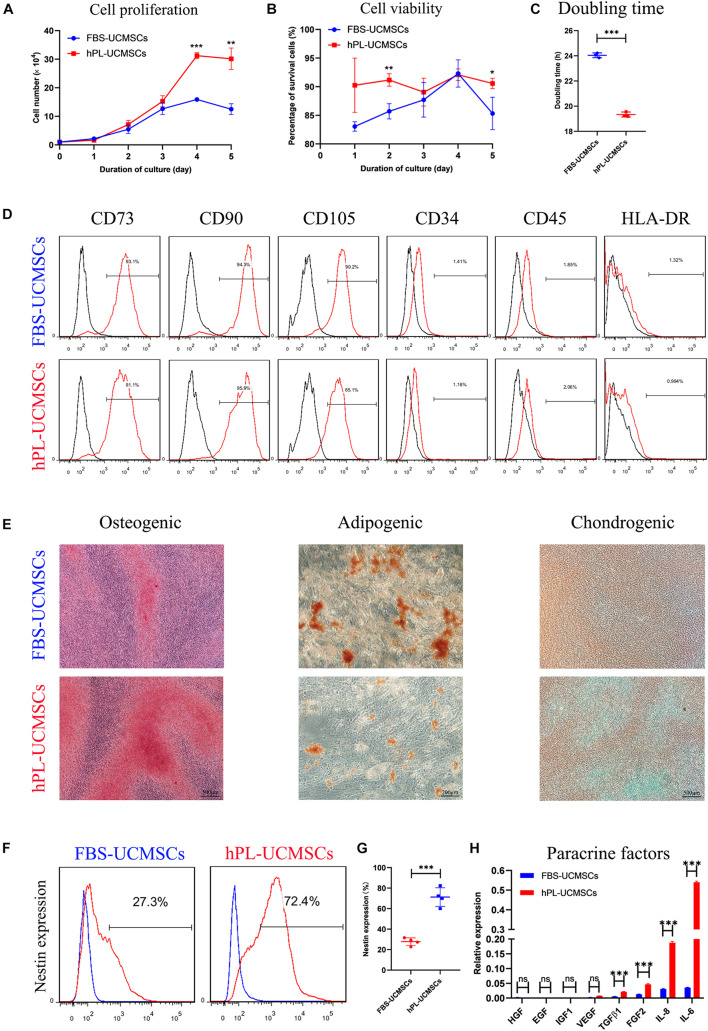
Growth supplement hPL maintain the stemness of UC-MSCs *in vitro* expansion compared with FBS. **(A)** Growth curves of P4 FBS-UCMSCs and hPL-UCMSCs were assessed by direct counting for 5 days. Three replicates were performed at each time point. **(B)** The cell viability of FBS-UCMSCs and hPL-UCMSCs in each point was investigated. **(C)** P4 UC-MSCs cultured with hPL represented lower doubling time (DT) than that in FBS, as analyzed by a formula: DT = *t* × [log 2/(log *N*_*t*_–log*N*_0_)], where *N*_*t*_ is the number of harvested cells, *N*_0_ is the number of seeded cells and t is the culture time. **(D)** The expressions of cell surface makers on P4 FBS-UCMSCs and hPL-UCMSCs were detected by flow cytometry. **(E)** Representative stained images showed the osteogenic, adipogenic, and chondrogenic potentials of P4 FBS-UCMSCs and hPL-UCMSCs, as confirmed by Alizarin red, Oil red O, and toluidine blue, respectively. Scale bars, 200 or 500 μm. The Nestin expression of FBS-UCMSCs and hPL-UCMSCs at P4 was analyzed by flow cytometry **(F)**; and the percentage of Nestin^+^ cells in the two groups was showed **(G)**. **(H)** The paracrine factors (growth factors and chemokines) that expressed by P4 FBS-UCMSCs and hPL-UCMSCs were compared using qPCR. Data are shown as mean ± SEM. *n* = 3–4. ^∗^*p* < 0.05, ^∗∗^*p* < 0.01, ^∗∗∗^*p* < 0.001.

Because P4 UC-MSCs are usually used for translational medicine applications, we aimed to investigate the influence of different culture supplements on P4 hPL-UCMSCs and FBS-UCMSCs. To do this, we measured cell proliferation, immunophenotype, and tri-lineage differentiation ability, as well as the levels of stem cell-related biomarker Nestin and paracrine factors. Cell counts indicated that on the fifth day of culturing, hPL-UCMSCs have a faster growth kinetics than FBS-UCMSCs, with higher proliferation especially on the fourth and fifth days of culture (Figure 2A). Moreover, the cell viability of hPL-UCMSCs were higher than FBS-UCMSCs during the second and fifth days of culture (Figure 2B). The population DT was significantly lower for hPL-UCMSCs compared with FBS-UCMSCs (19.34 ± 0.16 versus 24.04 ± 0.16 h, Figure 2C). Next, we performed immunophenotyping of hPL-UCMSCs and FBS-UCMSCs. At P4, more than 90% of these cells were positive for typical mesenchymal cell surface markers (CD73, CD90, and CD105), while measures of the expression of hematopoietic cell markers (CD34 and CD45) and HLA-DR were almost completely absent (Figure 2D). Next, we assessed the ability of hPL-UCMSCs and FBS-UCMSCs to differentiate into osteocytes, adipocytes, and chondrocytes at day 21 of culturing in the conditioned medium. Results indicate that hPL-UCMSCs exhibit better osteogenic and chondrogenic differentiation compared with FBS-UCMSCs. By contrast, only a small proportion of hPL-UCMSCs formed lipid granules, less than that in FBS-UCMSCs (Figure 2E); this indicates senescence of the cells prior to differentiating to adipocytes, but not osteocytes or chondrocytes. We next used flow cytometry to measure levels of cells positive for Nestin (Figure 2F). At P4, the proportion of Nestin in hPL-UCMSCs was much higher than in FBS-UCMSCs (71.25 ± 7.93 versus 27.75 ± 3.24%, Figure 2G). Finally, the function of tissue repair is associated with MSCs-secreted paracrine factors after MSCs transplantation. We used qPCR to analyze levels of HGF, EGF, IGF1, VEGF, TGF-β1, FGF2, IL-6, and IL-8; at P4, all levels were numerically higher for hPL-UCMSCs than FBS-UCMSCs, especially IL-6 and IL-8 (Figure 2H).

### Establishment of Animal Model to Mimic Bronchopulmonary Dysplasia Syndrome in Newborn Humans

**FIGURE 3:**
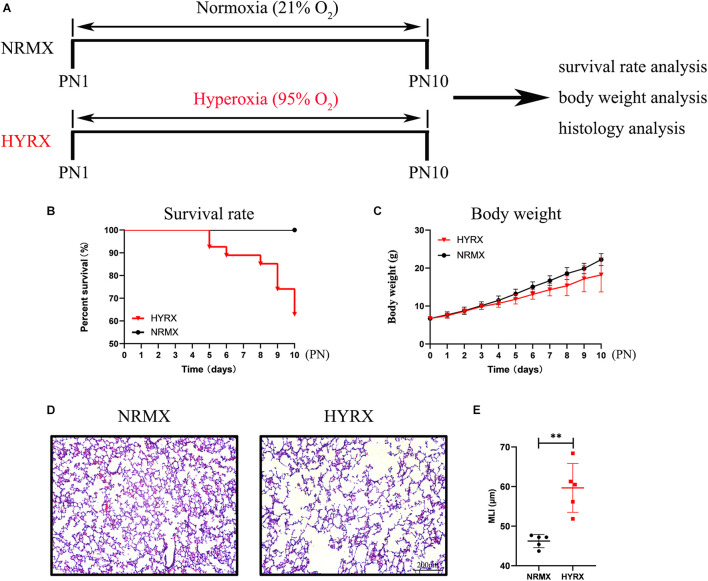
Schematic of protocols used for rat BPD establishment, and survival rate, body weight, and lung histology analysis. **(A)** Newborn rat pups were exposed to hyperoxia (HYRX; 95% O_2_) for 10 days during PN1 to PN10; rat pups that remained in normoxic (NRMX) conditions (21% O_2_) were compared as control group. During PN1 to PN10, the survival rate **(B)** and body weight **(C)** in each point were recorded. Rat lung tissue were harvested at PN10 and lung sections were stained with H&E **(D)**. Scale bars, 200 μm. Quantification of mean linear intercept (MLI) in each group represents a surrogate of average air space diameter **(E)**. Data are shown as mean ± SEM. *n* = 5–10. ^∗∗^*p* < 0.01.

As shown in Figure 3A, newborn rat pups were randomly assigned to either the NRMX or HYRX group, and from PN1 to PN10 were exposed to either Normoxia (21% O_2_) or Hyperoxia (95% O_2_), respectively. At PN10, their survival rate, body weight and lung histology were assessed to evaluate the applicability of the BPD rat model. The survival rates showed that newborn rat pups in the HYRX group developed difficulty breathing and gradually died after PN4 (HYRX: 63% survival, NRMX: 100% survival, Figure 3B); however, body weight was similar between the HYRX and NRMX groups in the first 4 days, with the HYRX group showing a trend toward decreased body weight after PN4 (Figure 3C). Next, the left lung tissues of both groups were collected at PN10. The HYRX group demonstrated a histological pattern reminiscent of human BPD, characterized by severe impairment of alveolar growth, large airspaces, and incomplete alveolar septation (Figure 3D), and this was reflected in elevated MLI values compared with the NRMX group (59.66 ± 5.53 versus 46.23 ± 1.49 μm, Figure 3E).

### Human Platelet Lysate-Human Umbilical Cord-Derived Mesenchymal Stromal Cells Are More Effective Than Fetal Bovine Serum Umbilical Cord-Derived Mesenchymal Stromal Cells for Alleviation of Bronchopulmonary Dysplasia Model

**FIGURE 4:**
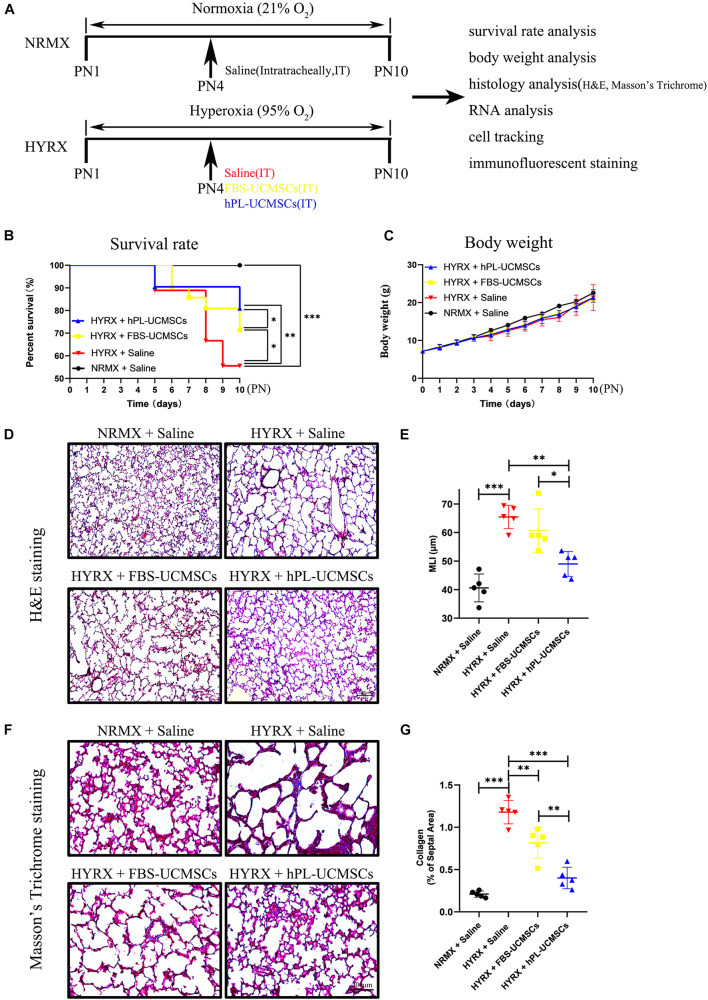
Superior therapeutic effects of hPL-UCMSCs versus FBS-UCMSCs on lung restoration in BPD model. **(A)** Schematic of protocols used for model establishment, administration of saline and UC-MSCs, and function analysis. During PN1 to PN10, the survival rate **(B)** and body weight **(C)** in each point were recorded among saline and UC-MSCs (FBS-UCMSCs or hPL-UCMSCs) treatment group. Rat lung tissue were harvested at PN10 and lung sections were stained with H&E **(D)**. Scale bars, 200 μm. Quantification of mean linear intercept (MLI) in each group represents a surrogate of average air space diameter **(E)**. Collagen deposition of lung sections were assessed by staining for Masson’s trichrome **(F)**. Scale bars, 200 μm. Collagen deposition was used as surrogate of fibrosis and was reported as percent of septal area **(G)**. Data are shown as mean ± SEM. *n* = 5–10. ^∗^*p* < 0.05, ^∗∗^*p* < 0.01, ^∗∗∗^*p* < 0.001.

As shown in Figure 4A, newborn rat pups were randomly assigned into four groups: NRMX + Saline, HYRX + Saline, HYRX + FBS-UCMSCs, and HYRX + hPL-UCMSCs. Newborn rat pups were exposed to normoxia (NRMX) or hyperoxia (HYRX) from PN1 to PN10. Saline, FBS-UCMSCs, and hPL-UCMSCs were injected intratracheally at PN4. Survival rate, body weight, histology, RNA, cell tracking, and immunofluorescent staining were analyzed at PN10.

The survival rates were significantly reduced in the HYRX + Saline group compared with the NRMX + Saline group, HYRX + FBS-UCMSCs group, or HYRX + hPL-UCMSCs group (55.56 versus 100, 71.43 and 80.95%, respectively; Figure 4B). Importantly, rats with hPL-UCMSCs treatment had higher survival rates compared to those with FBS-UCMSCs treatment (Figure 4B). However, there were no clear group differences in body weight, although the body weight in the three HYRX groups appears slightly lower than in the NRMX + Saline group from PN4 to PN10 (Figure 4C). These results suggest that hPL-UCMSCs and FBS-UCMSCs transplantation does not affect body weight. Next, the results of H&E and Masson’s Trichrome analyses showed that hPL-UCMSCs treatment alleviates lung histological pattern compared with the HYRX + Saline or HYRX + FBS-UCMSCs groups, with evident improvement in lung architecture and fibrosis (Figures 4D–G). Specifically, animals treated with hPL-UCMSCs presented with dramatically improved alveolarization and almost completely restored lung architecture compared with the HYRX + Saline group or the HYRX + FBS-UCMSCs group (Figure 4D); this difference was also reflected in significantly reduced MLI values in the animals treated with hPL (49.00 ± 3.89 versus 65.45 ± 3.64, and 60.65 ± 6.84 μm, respectively, Figure 4E). Collagen deposition (Masson’s trichrome stain) was used to measure the degree of lung fibrosis (Figure 4F), and lung tissue in the HYRX + Saline group had significantly higher levels of collagen deposition compared with the NRMX + Saline group (1.18 ± 0.12 versus 0.21 ± 0.03%); hPL-UCMSCs and FBS-UCMSCs treatment resulted in significantly decreased lung fibrosis, which was especially reduced in the HYRX + hPL-UCMSCs group (0.40 ± 0.11%, Figure 4G).

### Human Platelet Lysate-Human Umbilical Cord-Derived Mesenchymal Stromal Cells Transplantation Promotes Better Cell Retention in Lung Tissue Than Fetal Bovine Serum Umbilical Cord-Derived Mesenchymal Stromal Cells

**FIGURE 5:**
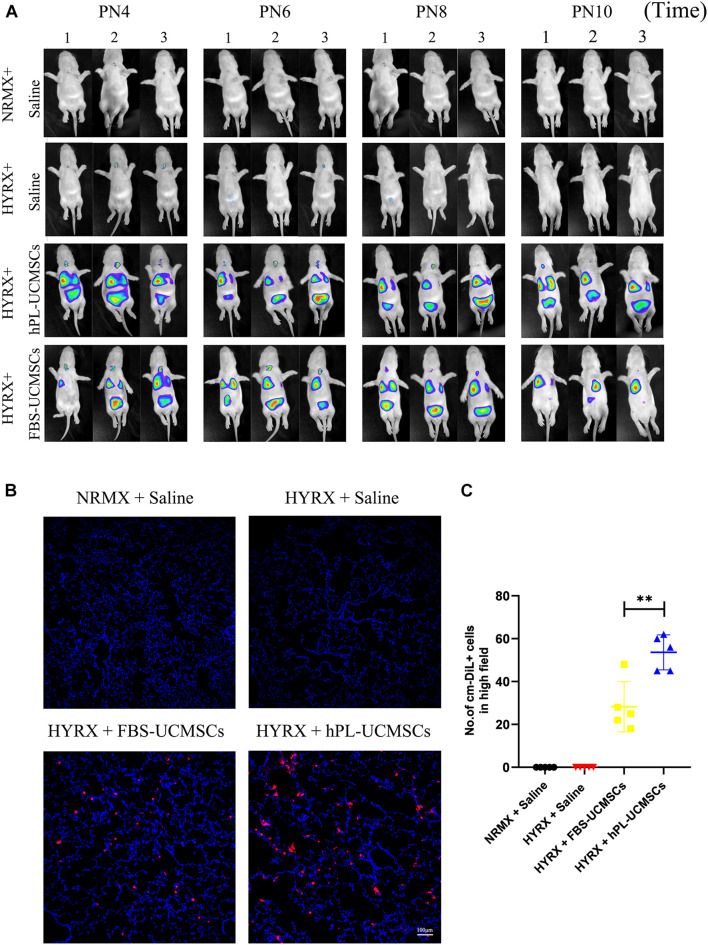
More hPL-UCMSCs were maintained in lung tissue than FBS-UCMSCs. **(A)** The biodistribution of hPL-UCMSCs and FBS-UCMSCs in rat lung during PN4 to PN10 was detected by In Vivo Imaging System. Rat lung tissue were harvested at PN10 **(B)**, and the count of administrated cm-DiL^+^ UC-MSCs in lung sections were evaluated from five random high field **(C)**. Scale bars, 100 μm. Data are shown as mean ± SEM. *n* = 5. ^∗∗^*p* < 0.01.

Our next analyses evaluated cell retention after hPL-UCMSCs and FBS-UCMSCs transplantation using Small Animal Imaging Technology and immunofluorescent staining. The biodistribution of hPL-UCMSCs and FBS-UCMSCs was measurable immediately in bilateral lung after transplantation at PN4. Following this we found that the cell counts in left lung of both groups gradually declined from PN4 to PN10, partly because of body metabolism (Figure 5A). Importantly, cell counts in left lung were clearly reduced in the HYRX + FBS-UCMSCs group compared with that in the HYRX + hPL-UCMSCs group; in particular, at PN10 the fluorescence signal was barely observable in left lung of the FBS-UCMSCs-treated group (Figure 5A). Next, the transplanted cells were stained with cm-DiL red dye, and the left lung tissues from the four groups were collected and fixed at PN10, after which cell retention was assessed by immunofluorescent staining (Figure 5B). The results indicated more cm-DiL+ cells in the HYRX + hPL-UCMSCs group than the HYRX + FBS-UCMSCs group (53.60 ± 7.28 versus 28.20 ± 10.44, Figure 5C). hPL-UCMSCs treatment increases capillary density and decreases inflammatory macrophage infiltration in lung tissue.

**FIGURE 6:**
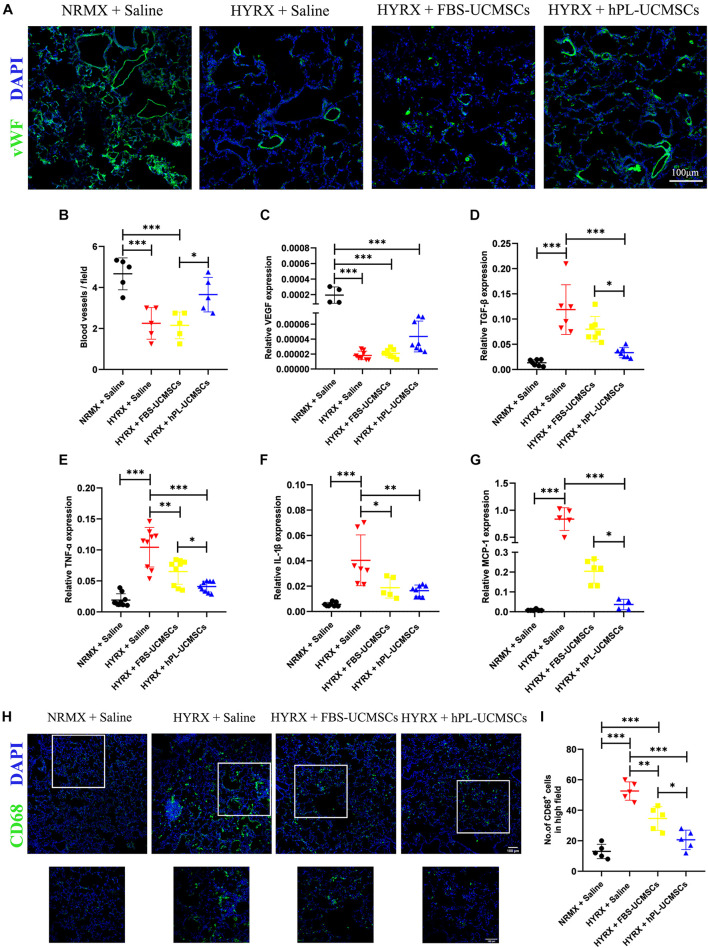
Human platelet lysate-human umbilical cord-derived mesenchymal stromal cells treatment rescued hyperoxia-induced loss of microvessels and suppressed macrophages-induced inflammatory response. Microvessels in lung sections were analyzed by staining with von Willebrand factor (vWF) at PN10 **(A)**; vWF-positive vessels that smaller than 50 μm were counted in five random views **(B)**. Scale bars, 100 μm. **(C)** The expression of vascular endothelial growth factor (VEGF) in lung tissue was analyzed by qPCR. **(D)** Rat TGF-β is related to lung fibrosis, so the TGF-β expression in treatment group was analyzed. TNF-α **(E)** and IL-1β **(F)** are the major factors in lung inflammation post-BPD, and their expressions were analyzed. **(G)** Monocyte chemoattractant protein-1 (MCP-1) can recruit monocytes from blood vessel to lung tissues, the MCP-1 expression was also evaluated. CD68^+^ inflammatory macrophages in the lung tissue at PN10 post-BPD were determined under fluorescence microscopy **(H)**; and calculated from five random fields of view for each experiment using a double-blind method **(I)**. Scale bars, 100 μm. Data are shown as mean ± SEM. *n* = 5–10. ^∗^*p* < 0.05, ^∗∗^*p* < 0.01, ^∗∗∗^*p* < 0.001.

To determine the effect of HYRX exposure on pulmonary vessel number, we assessed vWF staining in lung sections of rat pups at PN10 (Figure 6A). Compared with the NRMX + Saline group, HYRX exposure with saline treatment (HYRX + Saline group) led to significant loss of small vessels, which were less than 50 μm diameter (4.67 ± 0.70 versus 2.25 ± 0.69 blood vessels/field). In contrast, hPL-UCMSCs treatment (HYRX + hPL-UCMSCs group), but not FBS-UCMSCs treatment (HYRX + FBS-UCMSCs group), clearly helped maintain these vessels (3.65 ± 0.75 and 2.15 ± 0.58 blood vessels/field, respectively, Figure 6B).

We next collected the total RNA from lung tissues at PN10. The results of qPCR showed that rat VEGF expression was significantly reduced in the three HYRX-exposed groups when compared with the NRMX + Saline group; however, there was no significant difference between hPL-UCMSCs and FBS-UCMSCs groups (Figure 6C). Rat TGF-β expression in lung tissue reflects levels of collagen deposition and fibrosis. Results of qPCR showed that TGF-β expression was significantly reduced after hPL-UCMSCs treatment compared with FBS-UCMSCs and saline treatment in the three HYRX-exposed groups (Figure 6D). Expression levels of inflammatory factors (TNF-α and IL-1β) were also analyzed by qPCR, and we found lower expression of TNF-α and IL-1β in the HYRX + hPL-UCMSCs group compared with the HYRX + Saline or HYRX + FBS-UCMSCs groups; this demonstrates that hPL-UCMSCs transplantation significantly reduces the inflammation state in lung at PN10 (Figures 6E,F). In addition, monocyte chemoattractant protein 1 (MCP-1) expression was also significant lower in the HYRX + hPL-UCMSCs group than in the other two HYRX-exposed groups (Figure 6G). This result is important because chemokine MCP-1 can recruit monocytes from peripheral blood vessel into damaged tissue to become macrophages. Therefore, we analyzed inflammatory macrophage infiltration in lung tissue at PN10 using immunofluorescent staining (Figure 6H). The results demonstrated that CD68^+^ macrophages are significantly lower in the FBS-UCMSCs and hPL-UCMSCs groups compared with the HYRX + Saline group, and are particularly reduced after hPL-UCMSCs treatment (Figure 6I).

**FIGURE 7:**
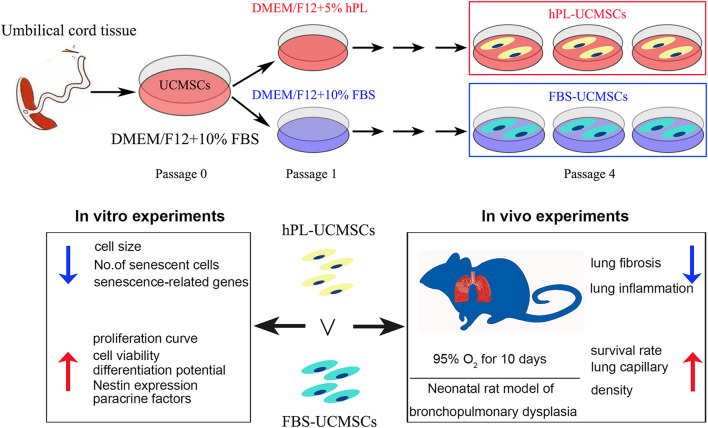
The overview of the work (graphical abstract).

In conclusion, this study demonstrated the superior effects of the new media supplement hPL over FBS on hUC-MSCs proliferation, senescence, differentiation, and paracrine factors secretion in *in vitro* expansion. Moreover, we demonstrated that hPL-UCMSCs are more effective than FBS-UCMSCs for lung repair in a rat BPD model (Figure 7).

## Discussion

Although FBS is a widely accepted standard, its use in clinical trials of MSCs therapy is discouraged by regulatory authorities due to its xenogenic origin. Recently, to search for xeno-free agents, hPL has been increasingly used as a substitute for FBS with several kinds of human stem cells, including bone marrow-derived MSCs (BM-MSCs) (Griffiths et al., 2013), ASCs (Phetfong et al., 2017; Søndergaard et al., 2017), periodontal ligament stem cells (PDLSCs) (Wu et al., 2017) and adult dental pulp stem cells (DPSCs) (Abuarqoub et al., 2015). However, there are few reports of hPL being used in hUC-MSCs culture and hPL-UCMSCs’ characteristics and function are under-investigated. In the current study, we not only examined the effect of hPL on hUC-MSCs proliferation, senescence, differentiation, and paracrine factors secretion, we also verified their therapeutic effect in a rat BPD model.

In this study, the cell culture supplement hPL was purchase from AventaCell BioMedical Corporation (UltraGRO^TM^-Advanced, GMP Grade, United States), and their instructions recommend that the 5% (v/v) of hPL provides optimal growth of MSCs in typical cell culture media. Griffiths et al. (2013) reported significantly greater cell growth in 5 and 2% hPL media formulations after 4 days of culture compared with FBS, and the 5% hPL formulation demonstrated significantly increased human bone MSC growth at day 7 and day 10 as well as reduced MSC DT compared with 10% hPL and FBS cultures. Additionally, Abuarqoub et al. (2015) showed that human DPSCs cultured with 5% hPL for 72 h resulted in the highest proliferation rates when compared with 10% hPL, 1% hPL, or 10% FBS. Therefore, only 5% hPL was used for comparing with FBS in our reported experiments. Moreover, the reason why we only used 5% hPL for UC-MSCs culture after P1, but not for the initial culture as Figure 1A depicted, was that hPL is not suitable for cells to climb out of umbilical cord tissue in the initial process. Therefore, it is too difficult to obtain enough P0 UC-MSCs in this way. In order to obtain similar amount and quality of P0 UC-MSCs for following serial experiments, we could only choose to use FBS for the initial culture. FBS contains abundant proteins that promote cell adhesion, such as vitronectin and fibronectin, which maybe promote the migration of UC-MSCs from umbilical cord tissue.

The cell morphologies of UC-MSCs cultured in 10% FBS and 5% hPL were clearly different. The 5% hPL culture resulted in UC-MSCs becoming more elongated and smaller than MSCs cultured in 10% FBS after P4, while UC-MSCs that were culture in FBS appeared wide and flat, and showed senescence at P10. The above results may be attributed to abundant growth factors and cytokines in hPL, which have a promotion of cell growth and proliferation, and keeping young. These results were similar to previous reports (Griffiths et al., 2013), which have found that MSCs maintained in 2 and 5% hPL formed distinct branching “network-like” morphologies and appeared more elongated than MSCs cultured in FBS; MSCs cultured with FBS exhibited a more flattened, spread-out fibroblast morphology and FBS cultures led to the emergence of many β-Gal-positive MSCs at P11. These findings suggest that hPL could be used for delaying senescence or even rejuvenating senescent MSCs (Vogl et al., 2013; Søndergaard et al., 2017).

Our findings also indicate that MSCs’ immunophenotype and differentiation differ after culturing with 5% hPL or 10% FBS. Not all previous research has found these differences; for example, Shansky et al. (2019) demonstrated a similar surface immunophenotype of adipose tissue MSCs (AT-MSCs) when cultured with hPL or fetal calf serum (FCS), including similar expression levels of CD73, CD90, and CD105, which gradually increased until P4 and were in proportions that generally exceeded 90%. In our study, CD73 and CD90 expression levels on FBS-UCMSCs and hPL-UCMSCs and CD105 expression on FBS-UCMSCs were close to 90%; however, CD105 expression on hPL-UCMSCs appeared lower (approximately 85%). Shansky et al. (2019) also reported slightly better adipogenic differentiation of AT-MSCs when cultured in hPL conditions compared with those cultured in FCS, and no considerable difference in osteogenic differentiation of AT-MSCs between hPL and FCS conditions. In contrast, our results demonstrated that hPL-UCMSCs show superior osteogenic and chondrogenic differentiation potential compared to FBS-UCMSCs, but there were no group differences in adipogenic differentiation. Our different pattern of results may reflect the use of FBS or FCS as control, and the possibility that senescent MSCs may be more easily differentiated into adipose cells.

Nestin is a class VI intermediate filament protein, which is usually expressed by stem-like cells, including neural stem or progenitor cells (Bernal and Arranz, 2018), tumor stem cells (Neradil and Veselska, 2015) and MSCs of various tissues (Yin et al., 2016; Lu et al., 2019; Liao et al., 2020). Nestin functions are associated with the self-renewal, proliferation, differentiation and migration of stem cells, and previous research reports that Nestin expression gradually decreases with increasing MSCs passage (Liu et al., 2020). In the current study we found higher Nestin expression in the hPL-UCMSCs group than FBS-UCMSCs at P4, which may be the cause of group differences in cell proliferation, senescence, and differentiation. In addition, hPL-UCMSCs expressed higher levels of paracrine factors than FBS-UCMSCs, including TGF-β1, FGF2, IL-8, and IL-6. It has reported that TGF-β1 released from MSCs leads to wound contraction and vessel formation (Jiang et al., 2020); other demonstrated that MSCs-released TGF-β1 promote CD4^+^CD25^+^Foxp3^+^ iTreg cells generation from human SLE PBMCs by to control SLE disease (Darlan et al., 2021). FGF2 gene regulates the production of basic fibroblast growth factors (bFGF). Narcisi et al. (2015) report that the WNT3A, in combination with FGF2, supports long-term expansion of human BM-MSCs (Narcisi et al., 2015). IL-6 is a pleiotropic cytokine with a wide range of functions. It can regulate the growth and differentiation of a variety of cells, regulate immune response, acute phase response and hematopoietic function, and play an important role in the body’s anti-infection immune response (Hunter and Jones, 2015). IL-8, an inflammatory chemokine with potent proangiogenic properties. Hou et al. (2014) reported that IL-8 enhances the angiogenic potential of hBM-MSCs by increasing VEGF production in part *via* the PI3K/Akt and MAPK/ERK signal transduction pathways. These above results may reflect how stem cell characteristics are affected by the use of hPL.

The rat BPD model employed in the current study was established according to a previously described protocol, but with some modified conditions. In the study of Willis et al. (2018) newborn FVB mice were exposed to hyperoxia (75% O_2_) for 7 days and then put into room air for another 7 days. Chang et al. (2014) reported that hyperoxic rat pups were raised in hyperoxic chambers (90% O_2_) from birth until PN14. In our study, newborn rat pups were exposed to either hyperoxia (95% O_2_) or normoxia (room air, 21% O_2_) from birth to PN10 in cages in an airtight Plexiglas chamber. Because of the higher concentration of O_2_ (95%), the rat pups largely died from PN5 onward. In response to the high death rate, we adjusted the time that rats were exposed to hyperoxia, which was reduced from 14 to 10 days. Additionally, the histological analysis of lung tissue in our study showed a pattern reminiscent of human BPD, characterized by severe impairment of alveolar growth, large airspaces, and incomplete alveolar septation. Because rat pups start to die at PN5, we administered hUC-MSCs or saline intratracheally at PN4.

## Conclusion

In conclusion, as depicted in my graphical abstract (Figure 7), we demonstrated that hPL is valuable as optimal supplement for UC-MSCs culture in research field. It will help to promote the wide use of hPL in the future industrial culture of UC-MSCs, and apply these MSCs for the treatment of BPD infant patients. Unfortunately, we did not make clear the difference of composition between hPL and FBS in this article, and some experiments need to do (e.g., mass spectrometry).

## Data Availability Statement

The original contributions presented in the study are included in the article, further inquiries can be directed to the corresponding authors.

## Ethics Statement

The studies involving human participants were reviewed and approved by the Maternal and Child Health Hospital of Longgang District. The patients/participants provided written informed consent to participate in this study. The animal study was reviewed and approved by the Animal Care and Use Committee of Shenzhen Beike Biotechnology Co., Ltd.

## Author Contributions

GL, YL, and DL: conception and design, manuscript writing, and collection and assembly of data. ZF, SW, DL, DC, and QO: collection and assembly of data, and data analysis and interpretation. ZT and GZ: data analysis and interpretation. XL, SX, and YL: administrative support. YL, JH, and ML: manuscript writing and final approval of the manuscript. All authors contributed to the article and approved the submitted version.

## Conflict of Interest

JH was employed by Shenzhen Beike Biotechnology Co., Ltd. (China) and provided the personnel and equipment support for this study. The remaining authors declare that the research was conducted in the absence of any commercial or financial relationships that could be construed as a potential conflict of interest. This study received funding from ML. JH and ML had the following involvement with the study design and final approval of the manuscript. ML once worked in Shenzhen Beike Biotechnology Co., Ltd., this study was started during that time and received funding from ML (but this fund was uniformly managed by Shenzhen Beike). Later, ML had resigned from Shenzhen Beike, and then JH continued to be responsible for subsequent experiments, he provided the personnel and equipment support for this study.

## Publisher’s Note

All claims expressed in this article are solely those of the authors and do not necessarily represent those of their affiliated organizations, or those of the publisher, the editors and the reviewers. Any product that may be evaluated in this article, or claim that may be made by its manufacturer, is not guaranteed or endorsed by the publisher.
